# Genetic analysis reveals four interacting loci underlying awn trait diversity in barley (*Hordeum vulgare*)

**DOI:** 10.1038/s41598-020-69335-x

**Published:** 2020-07-27

**Authors:** Biguang Huang, Daiqing Huang, Zonglie Hong, Swithin Omosuwa Owie, Weiren Wu

**Affiliations:** 1 Key Laboratory for Genetics, Breeding and Multiple Utilization of Crops, Ministry of Education, Fujian Agriculture and Forestry University, Fuzhou, 350002 Fujian China; 2 Fujian Collegiate Key Laboratory of Applied Plant Genetics, Fujian Agriculture and Forestry University, Fuzhou, 350002 Fujian China; 3 Fujian Key Laboratory of Crop Breeding by Design, Fujian Agriculture and Forestry University, Fuzhou, 350002 Fujian China; 4 Aquatic and Crop Resource Development, National Research Council of Canada, Saskatoon, SK S7N 0W9 Canada; 5 Department of Plant Sciences, University of Idaho, Moscow, ID 83844 USA

**Keywords:** Genetics, Plant sciences

## Abstract

Barley (*Hordeum vulgare*) awns contribute to grain yield, but the genetic basis of awn development remains largely unclear. Five barley lines differing in awn traits and row types were used to create four F_2_ populations. Genetic analyses revealed that four pairs of genes were involved in awn development: *A*/*a* (awnless/awned), *B*/*b* (awnless/awned), *H*/*h* (hooded/straight), and *L*/*l* (long/short). Of these four loci, *A*, *H* and *L* functioned on both central rows (CR) and lateral rows (LR) of the barley spikes, while *B* exhibited effect only on LR. *A* and *B* had duplicate effects on LR, and both showed dominant epistasis to loci* H* and *L,* whereas* H* was epistatic to *L.* Meanwhile, *A* and *B* were found to be genetically linked, with a row-type locus *V* located between them. The genetic distances of *A*-*V* and *B*-*V* were estimated to be 9.6 and 7.7 cM, respectively. Literature search suggested that *A*, *H* and *V* may correspond to the reported *Lks1*, *Kap1* and *Vrs1*, respectively, whereas *B* is a novel gene specifically controlling awn development on LR, designated as *Lsa1* for *lateral spikelet awnless 1*. The only barley homolog of wheat awn inhibitor gene *B1*, *HORVU2Hr1G077570*, is a potential candidate of *Lsa1*.

## Introduction

Barley (*Hordeum vulgare* L.) is one of the most important crops around the world. Awn is a needle-like extension from the top of lemma and contributes to grain yield and quality through photosynthesis^1,2^. Barley awns vary from awnless to awned with various shapes^3^. The awn shape can be hooded, crooked, leafy or straight. The hooded awns can be further distinguished into sub-types from normal hood-like to elevated hooded or subjacent hooded in shape. The straight awns can be single or branched, and long or short.

Barley spikelets are arranged on the central rows (CR) and lateral rows (LR) in a spike. Awn variation occurs on both central and lateral spikelets. Awn phenotypes on CR and LR are not always consistent. However, awns on LR and CR are usually phenotyped together as one trait. In some studies, the awn structure on LR is simply ignored^4^.

In barley, mutated genes of various morphological and developmental mutants collected from world-wide have been introduced into a common genetic background, the cultivar Bowman, to produce specific near isogenic lines (NILs) since 1985^5,6^. These NILs have been used to identify or isolate genes affecting morphological and developmental processes such as the floral bract gene *Hooded lemma1* (*Kap1*)^7^ and the spike row-type gene *Six-rowed spike1* (*Vrs1*)^8^. A number of additional genetic loci underlying awn development in barley have been identified and charaterized^9^, and their descriptions can be found at https://wheat.pw.usda.gov/ggpages/bgn/. The collection includes *Lks1* (for awnless, on 2HL), *sca1* (short crooked awns, on 3HS), *Kap1* (hooded awns, on 4HS), *sbk1* (subjacent hooded awns, on 2HS), *lks2* (short awns, on 7HL), *lks5* (short awns, on 4HS), *lks6* (short awns), *trp1* (triple awns, on 2HL), *lel1* (leafy lemma, on 2HL), and *lr* (reduced lateral spikelet appendage, considered as the same locus as *vrs1* on 2HL). Among these awn-related genes, only *Kap1* and *lks2* have been cloned and found to encode transcription factors^2,7^. The short awn gene *lks2* has been reported to be recessive epistatic to the hooded gene *Kap1*^10^. The relationships of other genes to these remain obscure.

In this study, we performed a thorough trait dissection and genetic analysis of four F_2_ populations with different awn type segregations. We proposed a four-gene model of interactions to interpret the awn phenotypic diversity, revealing a multi-layer relationship among these genes. We identified an LR-specific genetic locus, and showed that awns on CR and LR may have distinguishable genetic basis. Our findings lay a solid foundation for future cloning of the awn-related genes and improve our understanding of the genetic mechanism of awn development in barley.

## Results

### Awn inheritance in cross E21 × B19

**Figure 1:**
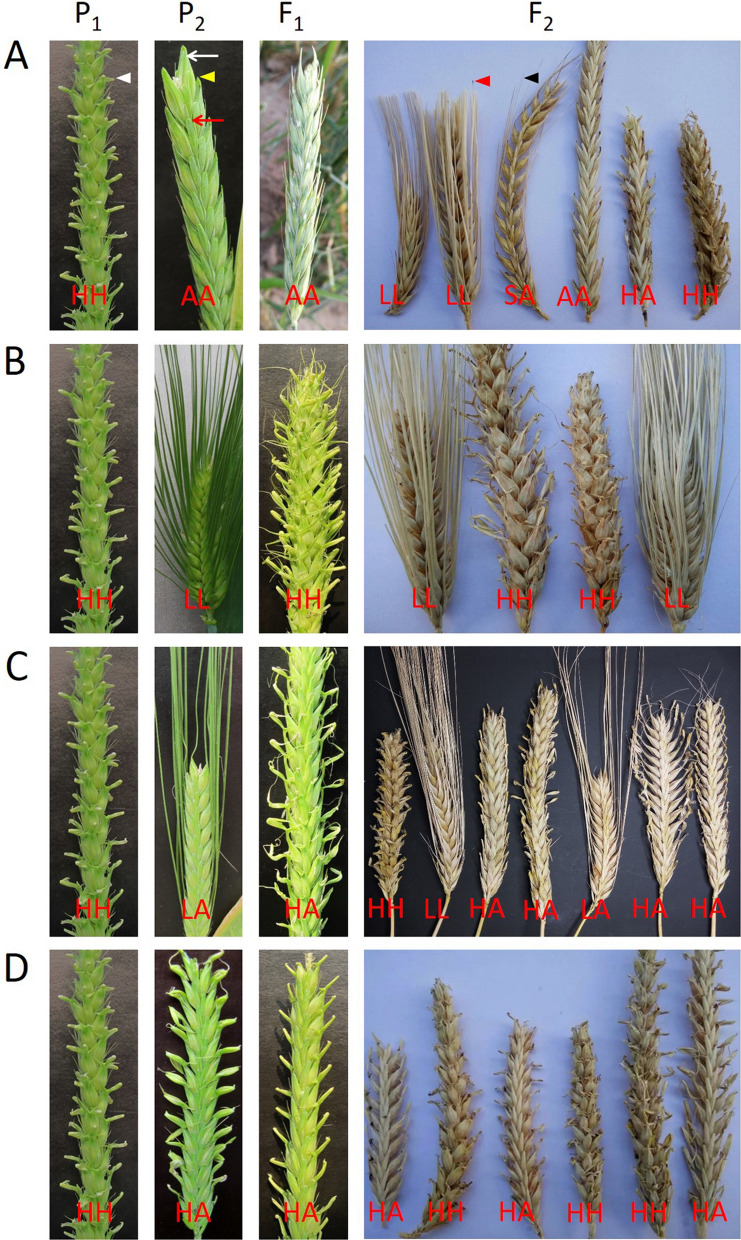
Awn phenotypes in the parents, F_1_ and F_2_ of four crosses. In the four crosses, P_1_ was E21, while P_2_ was B19 (**A**), E30 (**B**), B1 (**C**) or B33 (**D**). For each spike, the first and the second letters indicate the awn types of the central row (marked by white arrow) and the lateral row (red arrow), respectively. *H* hooded awn (denoted by white arrow head), *L* long awn (red arrow head), *S* short awn (black arrow head), *A* awnless (yellow arrow head).

**Figure 2:**
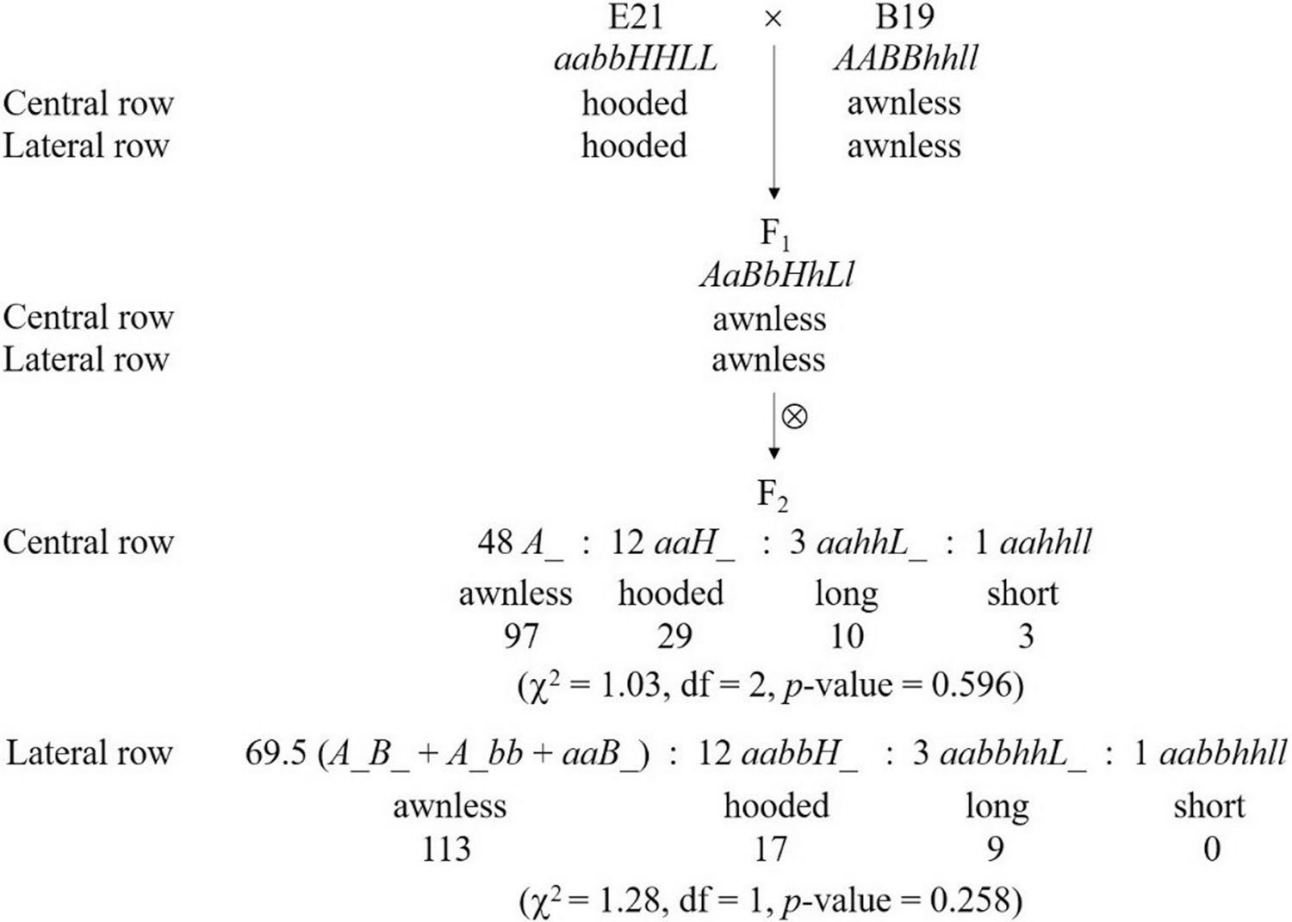
Awn inheritance in cross E21 × B19 as explained with the four-locus model. The observed number with each phenotype is shown underneath. For both CR and LR, the two phenotypes, long awn and short awn, were combined as one group in the Chi-squared test because there were not enough short-awn individuals. The expected segregation ratio on LR was determined by assuming that the RF between *A* and *B* was equal to the estimate 0.135, which used 1 degree of freedom.

In cross E21 × B19, the parent E21 developed hooded awns on both CR and LR, while B19 was awnless on both rows (Table 1, Fig. 1A). The F_1_ was awnless on both CR and LR, indicating that awnless was dominant on both CR and LR (Fig. 1A). A total of 139 F_2_ plants investigated were segregated into four phenotypes, namely, awnless, hooded awn, long awn and short awn (Fig. 1A), with the observed numbers being 97, 29, 10 and 3 on CR and 113, 17, 9 and 0 on LR, respectively (Fig. 2). Among them, awnless and hooded awn were the parental phenotypes, while long awns and short awns were two new phenotypes. These phenotypes could be classified into three hierarchical pairs of relative characters, namely, awnless vs. awned (including hooded awn, long awn and short awn), hooded awn vs. straight awn (including long awn and short awn), and long awn vs. short awn.

**Table 1:** Parental lines with different row types and awn phenotypes.

Parental line	Row type	Awn phenotype	
		Central row (CR)	Lateral row (LR)
B1 (Bowman)	Two-rowed	Long awn	Awnless
B19 (Bowman NIL)	Two-rowed	Awnless	Awnless
B33 (Bowman NIL)	Two-rowed	Hooded awn	Awnless
E21 (Fuding Heshang)	Six-rowed	Hooded awn	Hooded awn
E30 (Jinjiang Yuanmai)	Six-rowed	Long awn	Long awn

The segregation of awnless vs. awned among the 139 examind F_2_ plants was 97 vs. 42 on CR and 113 vs. 26 on LR. A Chi-squared test indicated that the segregations on CR and LR both followed the theoretical ratio of 3:1 (*p*-value = 0.19 and 0.11, respectively), suggesting that they were both controlled by a single locus with two different alleles. We named the two loci *A* (for CR) and *B* (for LR), respectively, of which alleles *A* and *B* caused awnless (dominant) and *a* and *b* produced awns (recessive). When considering CR and LR together, there were four phenotype combinations (CR/LR): awnless/awnless, awnless/awned, awned/awnless, and awned/awned, of which the observed number was 97, 0, 16 and 26, respectively. Obviously, the segregation ratio did not follow the Mendelian 9:3:3:1 ratio or any known segregation ratio of two interactive loci in the F_2_ generation. Fisher’s exact test indicated that the awnless/awned phenotype on CR and that on LR were significantly associated with each other (*p*-value = 1.558 × 10^−17^), suggesting that loci *A* and *B* were genetically linked. In addition, it can be seen from the segregation ratio that all CR-awnless plants were also LR-awnless, but not vice versa. This suggested that while *B* did not affect the awn phenotype on CR, *A* could affect the awn phenotype on LR. LR was awned only when *A* and *B* were homozygous for the recessive alleles, namely, *aabb*. In other words, *A* and *B* were equivalent in effect for awn phenotype on LR. Using Eq. (2), we obtained the maximum-likelihood estimate of the recombinant fraction (RF) between *A* and *B* to be 0.135, corresponding to a genetic distance of 13.8 cM. With this RF estimate, the awnless to awned ratio on LR was expected to be 113.0:26.0, which was exactly the same as the observed.

Within the awned subsets, the segregation of hooded awn vs. straight awn was 29 vs. 13 on CR and 17 vs. 9 on LR. A Chi-squared test indicated that the segregations of hooded vs. straight on CR and LR both followed the expected ratio of 3:1 (*p*-value = 0.48 and 0.37, respectively), suggesting that they were also controlled by a single locus each. Within the straight awn subsets, the segregation of long awn vs. short awn was 10 vs. 3 for CR and 9 vs. 0 for LR. A Chi-squared test was not suitable for testing the two segregation ratios because the sample sizes were too small. Nevertheless, the segregation on CR (10 vs. 3) was obviously very close to 3:1, and the segregations on CR and LR both showed a high *p*-value (= 0.873 and 0.178, respectively) of being 3:1 when the Chi-squared test was implemented approximately. Hence, it appeared that the variation of awn length was also controlled by a single locus for CR and LR, respectively. Noticeably, although the number of CR-awned plants and that of LR-awned plants were not equal (the former was larger than the latter), CR and LR always showed the same awned phenotype (hooded, long or short awns) in a plant with awned LR. This suggested that CR and LR were likely to be controlled by the same locus for the awned phenotypes. The plants displaying awned CR but awnless LR were likely to carry the dominant allele *B*. We designate the locus for hooded/straight awn as *H* (with alleles *H* for hooded awn and *h* for straight awn), and that for long/short awn as *L* (with alleles *L* for long awn and *l* for short awn), respectively.

Taken together, there were in total four loci involved in the awn variation in this cross: *A*, *B*, *H* and *L*. It has been inferred above that *A* functioned alone for awnless/awned on CR and together with *B* for awnless/awned on LR. According to the hierarchical relationships among the characters and the segregation ratios in the F_2_ population, it could be further inferred that *A* and *B* were both epistatic to *H* and *L*, and *H* was epistatic to *L*. However, while *A* was epistatic on both CR and LR, *B* displayed epistasis only on LR. We thus established a four-locus model of the awn inheritance in this cross (Table 2), which well explained the experimental results (Fig. 2). In the next section, it will be demonstrated that the awn inheritance in three other crosses with E21 as a common parent could also be fully explained by this model, which in turn supports this model.

**Table 2:** A four-locus model of awn inheritance in cross E21 × B19.

Locus	Effect position	Allele and function	
		Dominant	Recessive
*A*	CR and LR	*A*, awnless	*a*, awned
*B*	LR only	*B*, awnless	*b*, awned
*H*	CR and LR	*H*, hooded awn	*h*, straight awn
*L*	CR and LR	*L*, long awn	*l*, short awn

*A* and *B* had duplicate effect on LR, *A* and *B* were dominant epistatic to *H* and *L*, *H* was dominant epistatic to *L*.

### Awn inheritance in other crosses

**Figure 3:**
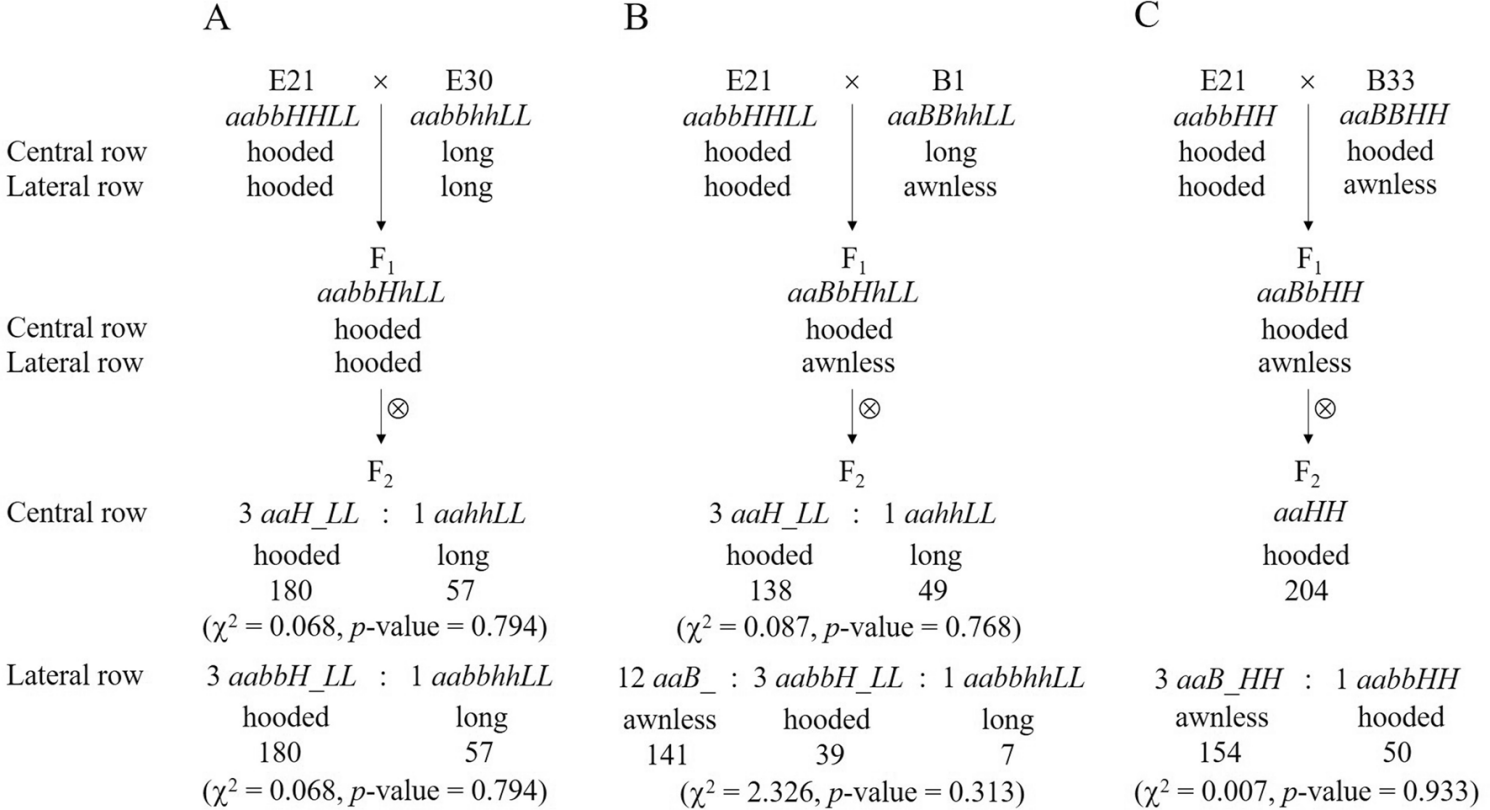
Awn inheritance in crosses E21 × E30 (**A**), E21 × B1 (**B**) and E21 × B33 (**C**) as explained with the four-locus model. The observed number with each phenotype is shown underneath.

In cross E21 × E30, the two parents showed hooded awns (E21) and long awns (E30) on both CR and LR, respectively (Table 1, Fig. 1B). As described above, the genotype of the common parent E21 was *aabbHHLL*. According to the proposed four-locus model (Table 2), it can be inferred that the genotype of E30 should be *aabbhhLL* and that the two parents differed at only one locus (*H* vs. *h*). As expected, the F_1_ (*aabbHhLL*) showed hooded awns on both CR and LR, and the F_2_ segregated into hooded awn and long (straight) awn plants with a ratio of 3:1 on both CR and LR (Figs. 1B, 3A).

In cross E21 × B1, compared with E21 (hooded awn on both CR and LR), the other parent B1 had long awns on CR and was awnless on LR (Table 1, Fig. 1C). Similarly, according to the four-locus model (Table 2), the genotype of B1 should be *aaBBhhLL*. Compared with the genotype of E21 (*aabbHHLL*), there were two loci showing allelic differences (*B* vs. *b* and *H* vs. *h*) between the two parents. As expected, the F_1_ (*aaBbHhLL*) displayed hooded awns on CR and awnless on LR, and the F_2_ segregated into hooded awn and long awn plants with a ratio of 3:1 on CR, and into awnless, hooded awn and long awn plants with a ratio of 12:3:1 on LR (Fig. 1C, 3B). To verify these results, we further investigated some F_3_ lines, each from a six-rowed F_2_ plant with hooded awns on CR but being awnless on LR. There were 35 F_3_ lines that showed hooded awns on CR but segregation of awnless vs. awned (hooded awns) on LR. These 35 F_3_ lines contained 753 LR-awnless plants and 240 LR-awned plants, following a 3:1 ratio (χ^2^ = 0.323, df = 1, *p*-value = 0.570), thus validating the existence of the *B* locus. In addition, by investigating 6 F_3_ lines that displayed segregation of hooded awn vs. straight long awn on CR and segregation of awnless vs. hooded awn vs. straight long awn on LR, we found that the segregations on CR (175 hooded : 51 long) and LR (170 awnless : 40 hooded : 16 long) followed the ratios of 3:1 (χ^2^ = 0.590, df = 1, *p*-value = 0.442) and 12:3:1 (χ^2^ = 0.398, df = 2, *p*-value = 0.820), respectively. The segregation on LR validated the epistasis of locus *B* to *H* and *L* and that of *H* to *L*.

In cross E21 × B33, while E21 showed hooded awn on both CR and LR, the other parent B33, displayed hooded awns on CR and awnless on LR (Table 1, Fig. 1D). Based on the four-locus model (Table 2), the genotype of B33 should be aaBBHH**, where the allele of locus *L* cannot be inferred from the phenotype of B33. As the genotype of E21 was *aabbHHLL*, B33 differed from E21 at locus *B*. It is noted that the hooded awn allele *H* existed in both parents. This means that the phenotype of straight awn (either long awn or short awn) could not appear because *H* was epistatic to *L*. Hence, locus *L* could be ignored and only one locus had allele difference (*B* vs. *b*) between the two parents. As expected, the F_1_ showed hooded awns on CR but awnless on LR, while the F_2_ showed hooded awns on CR in all plants but segregated into awnless and hooded awns on LR in a ratio of 3:1 (Fig. 1D, 3C).

### Genetic linkage between awnness and row type

Spikelets are arranged in two rows and six rows in a spike in barley. The common parent E21 in this study was six-rowed, while parents B1, B19 and B33 were two-rowed. In the three crosses E21 × B1, E21 × B19 and E21 × B33, their F_1_ hybrids were all two-rowed. Their F_2_ populations segregated into two-rowed vs. six-rowed plants with a ratio of 130 vs. 57, 106 vs. 33 and 154 vs. 50, respectively, which all followed the 3:1 ratio (*p*-value = 0.10, 0.81 and 0.94, respectively). These results suggested that the difference between E21 and other parental lines in row type was controlled by a single locus (denoted as *V*) with the two-rowed allele (*V*) being dominant to the six-rowed allele (*v*).

Genetic linkage between the awnless gene and row-type gene has been reported before^11^. To verify this, we examined the linkage of loci *A* and *B* with *V*. In the cross E21 × B19, the two-rowed parent B19 was awnless on both CR and LR, and the six-rowed parent E21 was awned (hooded awn) on both CR and LR (Table 1). Based on this, the genotypes of B19 and E21 for these three loci should be *VVAABB* and *vvaabb*, respectively. In the F_2_ generation, the combination of row type and awn on CR segregated into four types: two-rowed with CR-awnless (*V_A*_), two-rowed with CR-awned (*V_aa*), six-rowed with CR-awnless (*vvA_*), and six-rowed with CR-awned (*vvaa*). The segregation did not follow the expected Mendelian 9:3:3:1 for two independent loci, with much less recombinant phenotypes (*V_aa* and *vvA_*) in proportions (Table 3), suggesting that the two loci were genetically linked. Using Eq. (1), the RF between *A* and *V* was estimated to be 0.095.

**Table 3:** Linkage of loci *A* and *B* with locus *V*.

Cross	Linkage	Genotype segregation in F_2_				RF
E21 × B19	*V*-*A*	*V_A_* (95)	*V_aa* (11)	*vvA_* (2)	*vvaa* (31)	0.095
E21 × B19	*V*-*B*	*aaV_B_* (11)	*aaV_bb* (0)	*aavvB_* (5)	*aavvbb* (26)	0.076
E21 × B1	*V*-*B*	*V_B_* (126)	*V_bb *(4)	*vvB_* (15)	*vvbb * (42)	0.103
E21 × B33	*V*-*B*	*V_B_* (138)	*V_bb* (16)	*vvB_* (16)	*vvbb* (34)	0.173

The observed number of each phenotype is shown in parenthesis.

As demonstrated above that loci *A* and *B* were genetically linked. Therefore, *B* should be also linked with *V*. To estimate the RF between *B* and *V*, the association between row type and awnness on LR was analyzed. However, since *A* also affected awn phenotype on LR, plants with *V_B*_ genotypes could not be correctly identified when allele *A* was present. Thus, only CR-awned plants, which had the homozygous recessive genotype at locus *A* (i.e., *aa*), were suitable for analysis. There were 42 CR-awned plants in the F_2_ of E21 × B19, which segregated into 11 two-rowed plants with LR-awnless (*aaV_B*_), 0 two-rowed plants with LR-awned (*aaV_bb*), 5 six-rowed plants with LR-awnless (*aavvB_*) and 26 six-rowed plants with LR-awned (*aavvbb*) (Table 3). Using Eq. (1), the RF between *B* and *V* was estimated to be 0.076.

**Figure 4:**
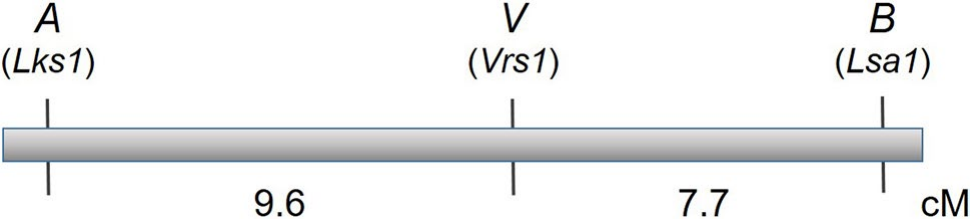
Linkage map of loci *A*, *B* and *V*. The genetic distances are in cM calculated from recombinant fractions (RF) using Kosambi’s mapping function.

Thus, we have obtained the estimates of RF as *r*_ab_ = 0.135 between *A* and *B*, *r*_av_ = 0.095 between *A* and *V*, and *r*_bv_ = 0.076 between *B* and *V*. As *r*_ab_ > *r*_av_ and *r*_bv_, and *r*_av_ + *r*_bv_ = 0.171, which was close to the estimate 0.135, it is likely that *V* is located between *A* and *B* (Fig. 4). If we take 0.171 as the RF estimate between *A* and *B*, the expected numbers of LR-awnless plants and LR-awned plants in the F_2_ of cross E21 × B19 will be 115.1 and 23.9, which are still very close to the observed numbers (113 and 26). This suggests that the inferred linkage order of the three loci is appropriate.

In addition, we also estimated the RF between *B* and *V* using the F_2_ populations of E21 × B1 and E21 × B33 because they did not contain allele *A*. The observed numbers of *V_B*_, *V_bb*, *vvB_* and *vvbb* in the F_2_ of E21 × B1 and those in the F_2_ of E21 × B33 were shown in Table 3. Using Eq. (1), the RF between *B* and *V* was estimated to be 0.103 from E21 × B1 and 0.173 from E21 × B33. These results verified the linkage between *B* and *V*, although the RF estimates were both larger than that obtained from E21 x B19, which might be due to the influence of different genetic backgrounds.

## Discussion

Previous studies have identified several genetic loci underlying awn development in barley^2,7,9^. However, the interactions among the genes and the genetic mechanism of awn development are still largely unknown. In previous observations, the phenotypes of awns on CR and LR have been treated as one character, or the awn phenotypes on LR have simply being ignored. In this study, we phenotyped CR awns and LR awns as independent traits. This led to the discovery of a LR-specific awnless gene *B*. We dissected the complex awn phenotypic variations into four pairs of relative traits and proposed a four-locus model for the genetic control of awn development. The duplicate effect of *A* and *B* on LR and the hierarchical dominant epistasis among the four loci revealed in this study indicated a complex genetic network for awn development in barley.

The phenotype and inheritance of the awnless gene *A* and hooded-awn gene *H* in this study were consistent with those of known awnless gene *Lks1* and hooded-awn gene *Kap1*, respectively^7,12,13^. The parents B19 and B33 used in this study have been known to carry *Lks1* and *Kap1*, respectively^6^*.* Therefore, we propose that loci *A* and *H* from this study are probably *Lks1* and *Kap1,* respectively*.* It has previously been known that the row-type variation (two-rowed vs. six-rowed) in barley is controlled by the locus *Vrs1*/*vrs1*^8^, which is linked with the awnless gene *Lks1* at a distance of ~ 9.6 cM on chromosome 2H (https://www.nordgen.org/bgs/index.php?pg=bgs_show&docid=229). In this study, we demonstrated that the row-type variation in the parental lines used was also controlled by a single locus *V* with the two-rowed being dominant to the six-rowed, and the row-type locus was linked with locus *A* at a distance of ~ 9.6 cM, which was similar to the one reported previously. This evidence further supports the notion that loci *A* and *V* are the *Lks1* and *Vrs1* genes, respectively.

Apart from *Lks1*, a previously characterized locus *Lr1* is also known to affect awn development on both CR and LR^14^. However, its dominant allele promotes rather than inhibits awn development, just opposite to that of *Lks1*. Therefore, the two genes, *Lks1* and *Lr1*, should be different. The LR-specific awnless gene *B* was a novel locus identified from this study. We here designate it as *lateral spikelet awnless 1* (*Lsa1*). Previously, a recessive gene named *lr* was reported to specifically affect awn development on LR in barley, which causes LR awnless, while its dominant allele helps awn development^15^. Therefore, *lr* is different from *Lsa1*. In fact, *lr* was thought to be the same as the six-rowed gene *vrs1*^16^.

Recently, several papers reported the identification of the dominant awn inhibitor gene *B1* in wheat^17–21^. Huang et al. functionally characterized the *B1* gene product as a C2H2 zinc finger protein with ethylene‐responsive element binding factor‐associated amphiphilic repression (EAR) motifs, and identified a homologous gene annotated as *HORVU2Hr1G077570* in barley^18^. By blasting the *B1* gene sequence against the Morex reference genome of barley (IBSC_v2) from EnsemblPlants, we found that *HORVU2Hr1G077570* was the only *B1* homolog, with 66% identity, in the barley genome (Supplementary Fig. S1), which was located at 561,127,844–561,128,267 bp on chromosome 2H, ~ 90 Mbp upstream of *Vrs1* (Supplementary Table S1)^22^. In the barley DNA marker database (https://153.126.143.92/cgi-bin/gb4/map_viewer_v04.cgi), *Vrs1* is located at 79 cM between MLOC_20933 and MLOC_64395 on 2H, while the marker MLOC_6548.1 for *HORVU2Hr1G077570* is located at 58 cM, which is 21 cM upstream of *Vrs1* (Supplementary Table S2). This indicates that the relative location of *HORVU2Hr1G077570* to *Vrs1* is similar to that of *Lsa1* to *Vrs1* (Table 3). The identical dominant inhibition effects of *B1* and *Lsa1* on awn development and similar relative locations of the *B1* homolog and *Lsa1* to *Vrs1* make *HORVU2Hr1G077570* a potential candidate gene for *Lsa1*. However, while *HORVU2Hr1G077570* is the only homolog of *B1* in barley, *B1* is not the only homolog of *HORVU2Hr1G077570* in wheat. In fact, there are two groups of genes in wheat related to *HORVU2Hr1G077570*. HORVU2Hr1G077570 is classified in group A with its wheat orthologs on chromosomes 2A, 2B and 2D, while B1 is classified in group B with paralogs on chromosomes 4B and 4D^18^. Further experiments need to be done to determine if *HORVU2Hr1G077570* performs a similar function as *B1* in inhibition of awn development.

The hooded awn is an appendage to the lemma, which consists of a deformed floret at its center with two triangular leaf-like lemma wings. The mature hood characteristics has been described in detail by Stebbins and Yagil^13^. Previous studies have shown that the hooded awn phenotype is controlled by a single gene *Kap1*^7,14,23^. It has been revealed that *Kap1* belongs to the *Knox* gene family^7^, and the hooded lemma is associated with the presence of a 305-base pair duplication in intron 4 of the *Knox3* sequence^24^.

Several genes controlling awn length have been reported before, including *lks2*, *lks5* and *lks6*^2,6,14,25^. It remains to be determined if the *L* locus identified in this study is allelic to any of the known awn-length genes based on the available information. Interestingly, however, it was reported that the short-awn gene *lks2* shows recessive epistasis to *Kap1*^10^. This is opposite to locus *L*, which was hypostatic to *H* (*Kap1*), suggesting that *L* is different from *lks2*.

## Materials and methods

Five barley germplasms with distinct awn phenotypes, B1, B19, B33, E21 and E30, were used as parental lines for crosses (Table 1). B1 is the well-known cultivar Bowman^5^ and B19 (BW431, *Kap1* NIL) and B33 (BW491, Lks1.b NIL) were two near isogenic lines (NILs) of Bowman^6^. These lines were obtained from Dr. Brian Forster (International Atomic Energy Agency) and Dr. Jerry D. Franckowiak (North Dakota State University). B19 and B33 are known to carry *Lks1* and *Kap1*, respectively. E21 (Fuding Heshang) and E30 (Jinjiang Yuanmai) are landraces from Fujian Province, China.

E21 was used as a common parent to cross with the remaining four germplasms, resulting in four crosses including E21 × B19, E21 × B33, E21 × B1, and E21 × E30. The awn and row-type phenotypes of parents, F_1_ hybrids and F_2_ or F_3_ individual plants grown under the same conditions were recorded. The awn phenotypes on central rows (CR) and lateral rows (LR) were examined and recorded separately. Genetic analysis was performed for the observed data for each dissected trait and a genetic model was proposed to explain the awn phenotype segregation in the F_2_ populations studied. Chi-squared test was used to examine the degree of fit between the observed data and the theoretical values expected according to the proposed genetic model. Yates’s correction for continuity was adopted when only two groups were involved in the Chi-squared test.

The recombinant fraction (RF) between two linked loci was estimated based on F_2_ data using the maximum likelihood method, and the RF was converted into genetic distance using Kosambi’s mapping function^26^. For two non-interactive loci (*A-a* and *B-b*) linked in coupling phase with an RF of *r* between them, the segregation ratio of *A_B_*:*A_bb*:*aaB_*:*aabb* is expected to be (1 − *r*)^2^/2:*r*^2^/2:*r*^2^/2:(1 − *r*)^2^/2 in an F_2_ population. When the observed numbers of *A_B_*, *A_bb*, *aaB_* and *aabb* are *n*_1_, *n*_2_, *n*_3_ and *n*_4_, the maximum likelihood estimate of RF is^27^:

1 $$r = 1 - \sqrt {\frac{{m + \sqrt {m^{2} + 8nn_{4} } }}{2n}} ,$$

where *n* = *n*_1_ + *n*_2_ + *n*_3_ + *n*_4_, and *m* = *n*_1_ − 2*n*_2_ − 2*n*_3_ − *n*_4_. When dominant *A* and *B* are duplicate in effect, the F_2_ segregates into two phenotypes, namely, *A_B_* + *A_bb* + *aaB_* and *aabb*, of which the expected ratio is [1 − (1 − *r*)^2^/2]:(1 − *r*)^2^/2, and the observed numbers are *n* − *n*_4_ and *n*_4_, respectively. In this case, *r* can be easily estimated by solving equation (1 − *r*)^2^/2 = *n*_4_/*n*, which is the same as the maximum likelihood estimate:

2 $$r = 1 - 2\sqrt {\frac{{n_{4} }}{n}} .$$

## Conclusions

Four genetic loci (*A*, *B*, *H* and *L*) underlying awn development in barley have been identified and the genetic relationships among them have been established in this study. *B* represents a novel locus specifically controlling the awn phenotype on LR and is designated as *Lsa1* for *lateral spikelet awnless 1*, which could potentially be the wheat awn inhibitor *B1* homolog *HORVU2Hr1G077570*. The results of this study will facilitate further dissection of the complex genetic basis of awn development and awn diversity in barley.

## Supplementary information

Supplementary Figure 1.

Supplementary Table 1.

Supplementary Table 2.
