# Supplementary material for: Genetic analysis reveals four interacting loci underlying awn trait diversity in barley (Hordeum vulgare)
Source: Sci Rep. 2020 Jul 27;10:12535. doi: 10.1038/s41598-020-69335-x (PMC7385259; doi:10.1038/s41598-020-69335-x)
Supplement: Supplementary file 1 — Supplementary Figure 1. [file 41598_2020_69335_MOESM1_ESM.pdf]

Interactions of four genetic loci underlying awn diversity and linkage between awnness and row type in barley (*Hordeum vulgare*)

Biguang Huang, Daiqing Huang, Zonglie Hong, Swithin Omosuwa Owie, Weiren Wu

Supplementary Figure S1. *HORVU2Hr1G077570* alignment with wheat *B1*.

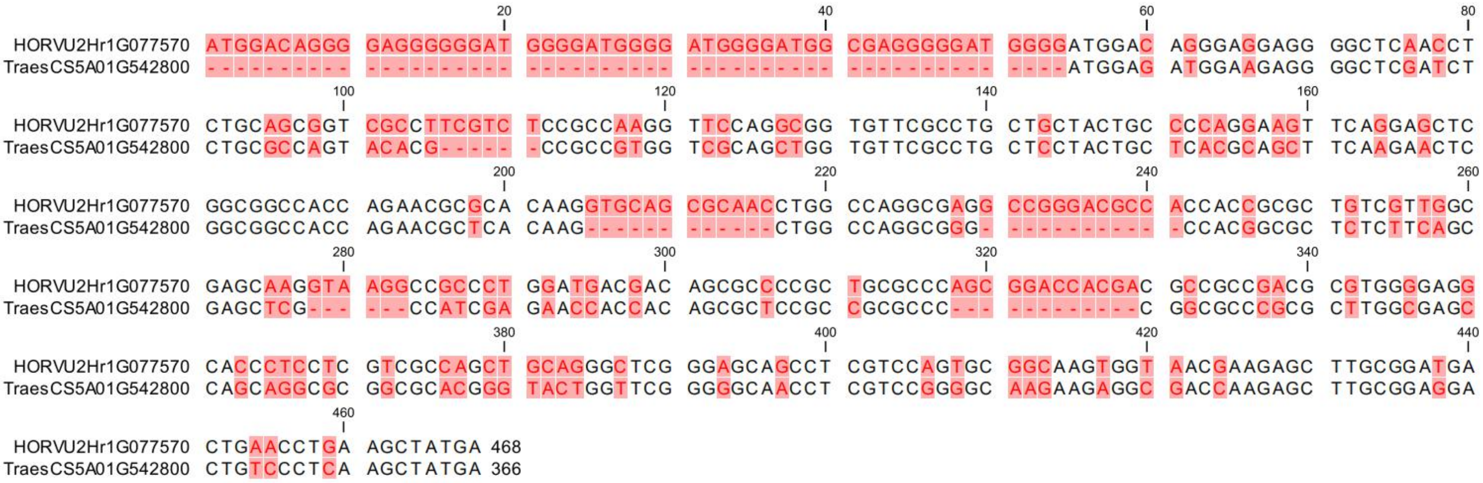

Huang, D. *et al*. Dominant inhibition of awn development by a putative zinc-finger transcriptional repressor expressed at the *B1* locus in wheat. *New Phytol.* **225,** 340–355 (2020).
